# Social Media Monitoring of Discrimination and HIV Testing in Brazil, 2014–2015

**DOI:** 10.1007/s10461-017-1753-2

**Published:** 2017-03-27

**Authors:** René Clausen Nielsen, Miguel Luengo-Oroz, Maeve B. Mello, Josi Paz, Colin Pantin, Taavi Erkkola

**Affiliations:** 1 UN Global Pulse, New York, USA; 2 UNAIDS, Brasília, Brazil; 3 Department of STI, AIDS and Viral Hepatitis, Brazilian Ministry of Health, Brasília, Brazil; 4 UNAIDS Secretariat, 20 Avenue Appia, 1211 Geneva, Switzerland

**Keywords:** Social media, Big data, HIV testing, Discrimination, Perceptions, Advocacy

## Abstract

Big data can be used to assess perceptions about public health issues. This study assessed social media data from Twitter to inform communication campaigns to promote HIV testing and reduce discrimination related to HIV/AIDS or towards key populations to the HIV epidemic, and its potential utility to evaluate such campaigns through HIV testing uptake. Tweets from Brazil were collected from January 2014 to March 2015 and filtered by four categories of keywords including discrimination, HIV prevention, HIV testing, and HIV campaigns. In total over 100,000 geo-located tweets were extracted and analyzed. A dynamic online dashboard updated daily allowed mapping trends, anomalies and influencers, and enabled its use for feedback to campaigns, including correcting misconceptions. These results encourage the use of social networking data for improved messaging in campaigns. Clinical HIV test data was collected monthly from the city of Curitiba and compared to the number of tweets mapped to the city showing a moderate positive correlation (r = 0.39). Results are limited due to the availability of the HIV testing data. The potential of social media as a proxy for HIV testing uptake needs further validation, which can only be done with higher frequency and higher spatial granularity of service delivery data, enabling comparisons with the social media data. Such timely information could empower early response immediate media messaging to support programmatic efforts, such as HIV prevention, testing, and treatment scale up.

**Electronic supplementary material:**

The online version of this article (doi:10.1007/s10461-017-1753-2) contains supplementary material, which is available to authorized users.

## Introduction

Of the 37 million people living with HIV in the world, 15 million do not know their HIV-positive status [1]. Ending the AIDS epidemic is in sight, and is being set as a target for year 2030. Key to reaching the target is testing and diagnosing people living with HIV, which will enable them to access treatment, and in turn improve their quality of life and reduce new infections. While, globally, the number of new HIV infections has systematically been decreasing since its peak in 1997, new infections among one of the most affected populations, gay men and other men who have sex with men, continue to expand in most countries [2]. One of the greatest bottlenecks to reach the 2030 global goal is stigma and discrimination related to HIV and AIDS and specific populations that carry a disproportional burden of the epidemic. Stigma and discrimination hinder people’s access to health care services and their ability to remain in care. Monitoring and evaluation efforts in countries have traditionally focused on epidemiological, clinical and surveillance data [3]. Population level surveys have been used to measure people’s knowledge and behaviours, which have informed prevention efforts. However, due to their cost and timeliness (once every 3–5 years), there is a need to complement such data with more frequent, dynamic and granular data collection efforts. Use of big data, such as social media data is being explored as means to expose discrimination and other behavioural bottlenecks.

The United Nations has called for a Data Revolution that seeks to fill data gaps, decrease the data quality differences between rich and poor countries, and improve collaboration between old and new data producers and owners [4]. Big Data is an umbrella term referring to large amounts of digital data continually generated by the global population that can be leveraged to contribute to close these gaps and complement traditional data sources [5]. There is an increasing body of evidence of the use of Big Data to inform HIV/AIDS programs. Similar to findings in studies using web search data to track diseases such as influenza [6] and dengue fever [7], in US state internet search rates for the term “HIV” on Google were strongly correlated with data on new cases of HIV [8]. The top HIV-related searches from different provinces of South Africa [9] were identified, showing that search patterns and terms do vary by province, which suggested the need for tailored campaign messaging for each province.

Social media analysis has been used for understanding perceptions and concerns in public health issues and epidemiology [10], such as the introduction of new smoking products [11], parents’ perceptions on vaccination [12], contraception methods [13] or the Ebola epidemic [14]. HIV risk-related tweets were compared to HIV prevalence rates at the state and county levels in the US [15]. From more than 2 million geo-located tweets from the United States they found around 10,000 tweets that were relevant to HIV risk taking behaviour (i.e., drug use and sex) during the approximately 6-month period of the study. Using negative binomial regression, they found a significant positive relationship between the number of tweets pertaining to HIV risk behaviour and the official case reports. While there exists some caveats and bias in the content likely to be communicated on social media and the indirect link between HIV transmission and the risk factors being tracked online (sex and drugs usage) [16], social media for disease surveillance offers a high potential opportunity—in terms of speed, breadth and cost—as a complementary approach to traditional surveillance systems.

“Zero Discrimination campaign” was launched in 2014 by the Brazilian President Dilma Rousseff, in support of a new law that prohibits discrimination against people living with HIV. The campaign was run throughout the time of the World Cup to reduce barriers for HIV testing and promote HIV prevention efforts, which also motivated the timing of this study.

In this project, we have developed social media monitoring tools and methods to capture the Brazilian population’s perceptions of HIV and AIDS, HIV prevention, and discrimination towards HIV and AIDS and key populations in order to support programmatic HIV prevention activities in Brazil—including the UNAIDS’ “Zero Discrimination Campaign”. The initial premise of this study is that there is a connection between (a) HIV information and related campaign on anti-discrimination, and (b) how people respond and reflect in social media, and (c) how they use related services (HIV testing and treatment) in health facilities across the cities involved in the campaign and the World Cup. Therefore, two hypotheses were explored:

1. If the anti-discrimination campaign is working and having impact in social media, there will be growing number of tweets about discrimination—and the possibility to influence such conversation.
2. The more is Tweeted on HIV, the more people end up testing for HIV.

These hypotheses translated into the following research objectives for the benefit of the Brazilian campaign:

1. Monitoring how the anti-discrimination and prevention campaigns were accepted by the public in social-media messages, and test if such monitoring could be used (by The Brazilian Ministry of Health and UNAIDS) for adapting the campaigns in real-time.
2. Utilizing Twitter to infer HIV related indicators, by comparing time series of aggregates of tweets with clinical HIV testing data. This study focused in Curitiba because of the poor availability of the testing data from facilities in other cities across the country.

## Data and Methods

**Fig. 1:**
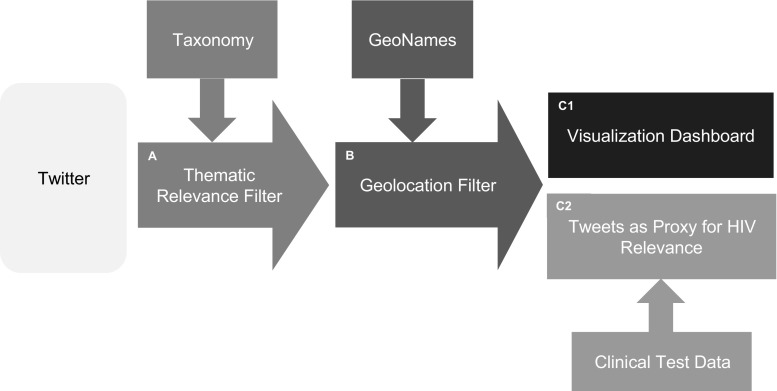
Processing framework: **a** First, tweets were filtered against a taxonomy consisting of 1966 keywords and phrases relevant to HIV & Discrimination. **b** Secondly, it was determined whether each tweet was written in Brazil (latitude and longitude from metadata) or written by a person from Brazil (self-reported user location). **c1** All tweets matching the taxonomy and with a relation to state capitals and World Cup host cities were included in the visualization dashboard. **c2** Statistics on tweets from Curitiba where compared against clinical data in the number of HIV tests performed

This research analysed all the public Tweets published between January 2014 and March 2015 in Brazil. The Twitter Firehose was accessed through the Datasift platform to filter out the relevant messages from the approximately 228 billion public tweets posted during the period studied. The data processing pipeline to filter the relevant HIV and discrimination related tweets developed in this project can be divided into two steps (Fig. 1):

### Taxonomy Filter

A Portuguese taxonomy consisting of 1966 keywords and phrases related to HIV/AIDS and discrimination was used to filter and extract the initial dataset for this study: if the text of a tweet contains any of the keywords and phrases of the taxonomy is considered relevant and included in our dataset. The taxonomy was structured around the four overarching themes of “Discrimination”, “HIV Prevention”, “HIV Testing”, and “HIV Campaigns”. The themes “Discrimination”, “HIV Prevention”, “HIV Testing” were subdivided into “positive” and “negative” reflecting tweets supporting non-discrimination or discriminatory tweets; those promoting or combating HIV preventive methods or behaviours; and messages stimulating or avoiding one’s knowledge of HIV serostatus through testing uptake.

The taxonomy was created by HIV campaigning and social media experts from the Brazilian Ministry of Health and UNAIDS Brazil. The initial taxonomy went as broad as possible to initially optimise the breadth of tweets gathered (recall), with each new iteration narrowing the scope by using negatives (e.g., “vencer” AND “preconceito” AND NOT “se o amor” to rule out tweets citing “Mas se a vida te der alguem melhor que eu, e se o amor vencer todos os preconceitos” from a popular song) and double clauses (e.g., ‘sexo’ AND ‘camisinha’) to reach a level where precision reached a previously determined acceptable level (at least 95% relevancy). This taxonomy allowed extracting a dataset consisting of 1,357,665 tweets. Both the taxonomy and the message identifiers to retrieve the database are available in the Supplementary Material.

### Tweets Geolocation

In order to limit the geographical scope to Brazil, a geolocation algorithm based on GeoNames data [17] was run as an initial location step over the 1,357,665 tweets. A tweet was deemed to be within the geographical scope of the project if its metadata contained a latitude-longitude combination from within the Brazilian borders or if the user’s profile listed a place in Brazil under “Location”. This process allowed us to extract 418,207 tweets that were used to provide overall Topic Trends and to identify Top Tweets for the full country. Using another GeoNames based filter, tweets that were written from within 50 kilometres of any of 27 World Cup host cities and state capitals were selected resulting in a final dataset with 100,133 tweets.

In addition, all tweets identified to be from the city of Curitiba were singled out (7546 tweets) in order to see if the HIV test data from all Curitiba clinics (public and private; primary health care, pregnant women, voluntary counseling and testing) were associated with the number of tweets written about HIV and AIDS and discrimination. Curitiba is the capital of a Southern Brazilian State Paraná, the eighth most populous city in Brazil (about 2 million people) and is one of the cities with most advanced AIDS response and centralised data collection mechanisms.

## Results

### Visualization Dashboard

**Fig. 2:**
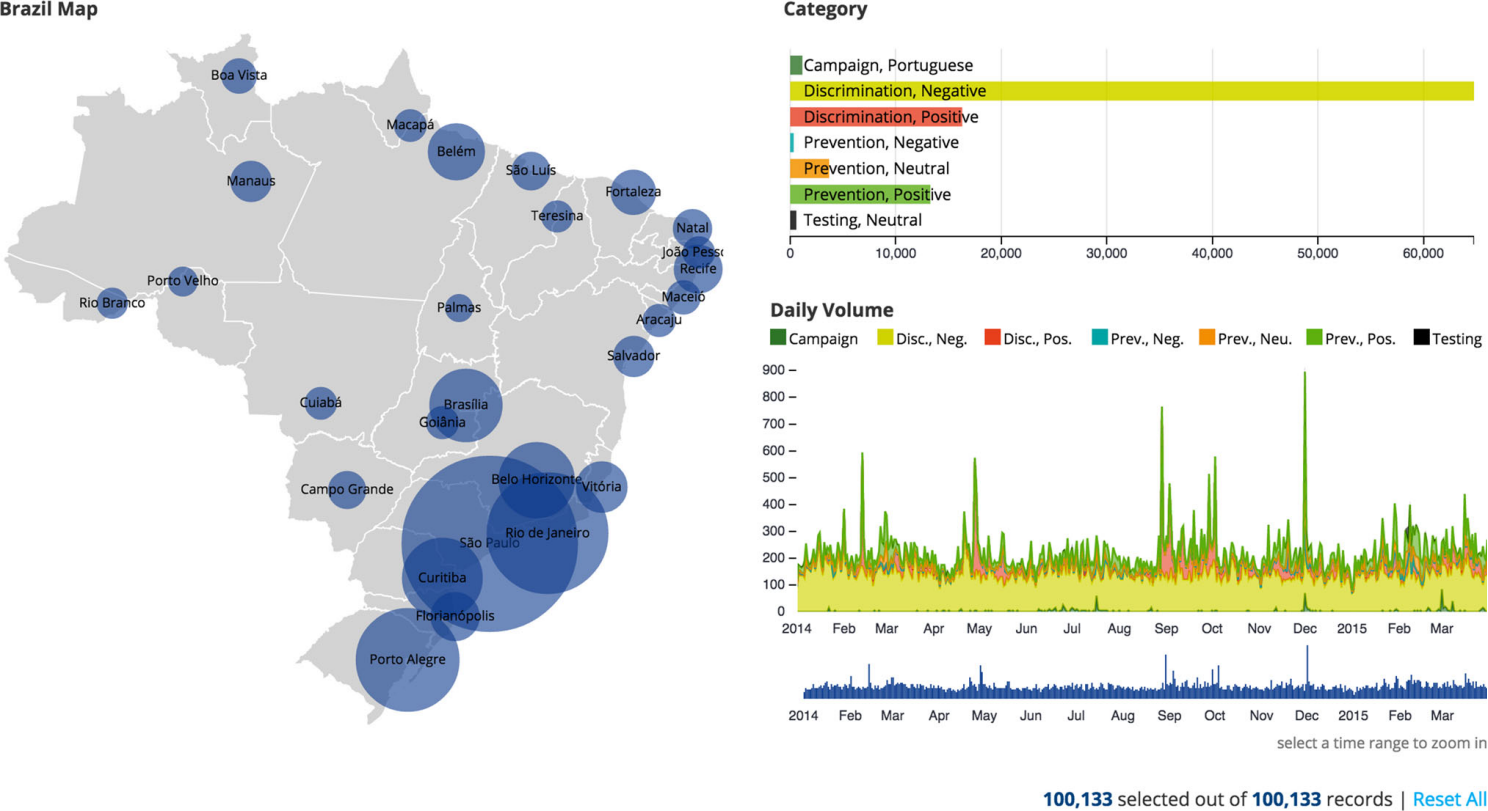
The main dashboard shows the 27 cities chosen for the study on a map with bubbles sized by number of tweets about HIV prevention, HIV testing, HIV campaigns and discrimination, a bar chart with topics and sentiment, and a time-series chart. There were more discriminatory tweets (65%) than in the other topics combined, and 21% of the tweets were from São Paulo

**Fig. 3:**
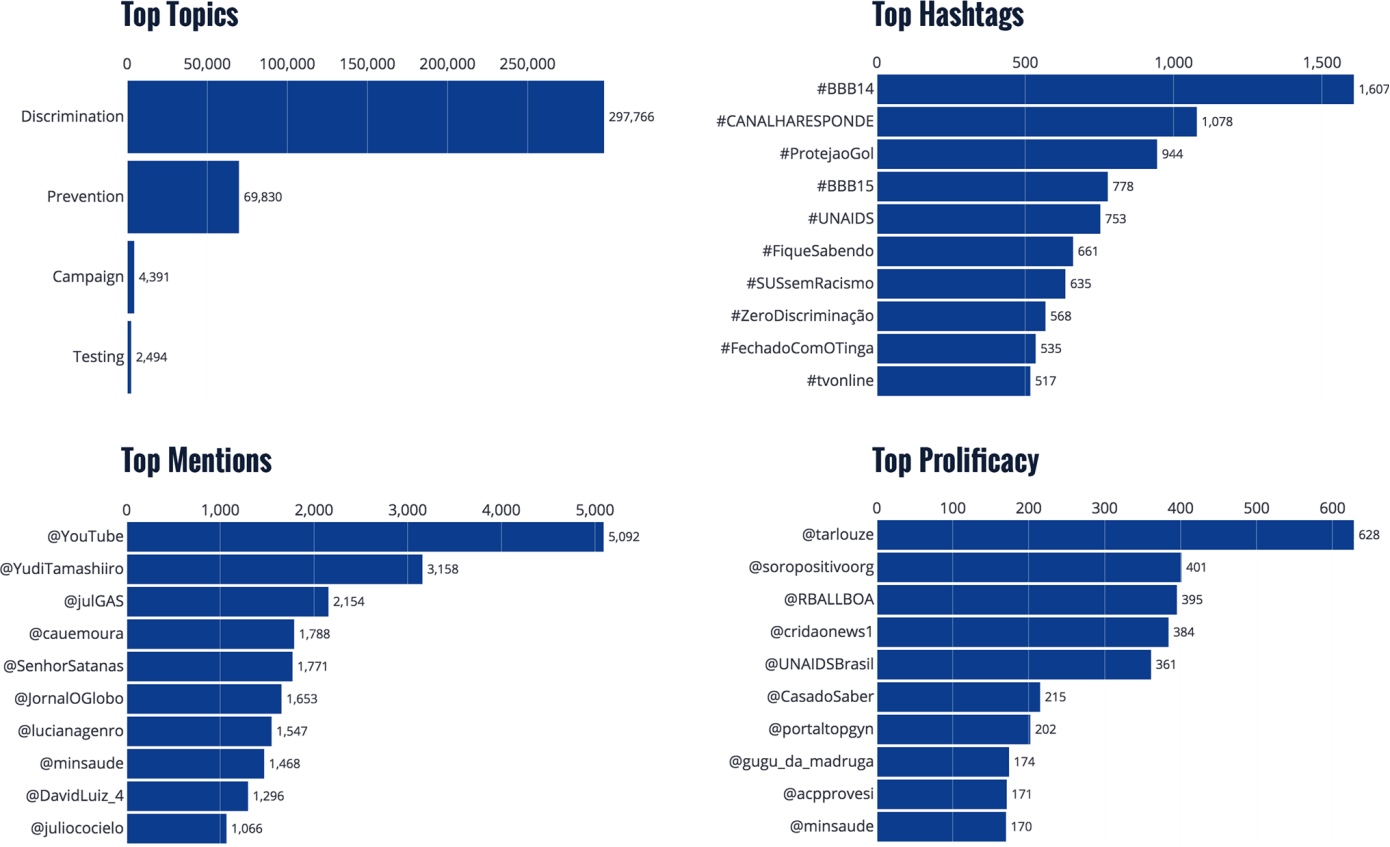
*Bar charts* show the accumulated overall topic distribution (*top left*), most used hashtags (*top right*), most mentioned accounts (*lower left*) and most prolific accounts (*lower right*)

In order to accomplish the first objective of this research and provide feedback to the campaign strategy, the extracted data and analysis were presented in an interactive web based dashboard that was updated daily with a morning update that could be assessed by the campaign organizers before their daily activities. The dashboard was designed to enable quick insights into trends and anomalies, showing interactive charts that allowed to disaggregate the data by time, city and topic (see Fig. 2). The dashboard also monitored the most relevant tweets in a rolling 7-day window and tweets written by most followed and influential users, allowing to easily reply and retweet [18], thereby increasing the likelihood of rapid feedback from the campaign organizers (Fig. 3).

The graphic illustrates that during the FIFA World Cup in Brazil (June–July) there was consistent volume of positive HIV prevention messages (campaign period) but no sharp peaks. September–October data shows higher activity in positive prevention messages, culminating to the first of December, the World AIDS Day. Negative discriminatory messages are observed throughout the period, with no great changes over time. The real time dynamic nature of the interactive visualization dashboard served as high level situation awareness tool, also providing for the local campaign managers and decision makers actionable and detailed information around key messages.

### Tweet Volume as a Proxy for HIV Testing Uptake

**Fig. 4:**
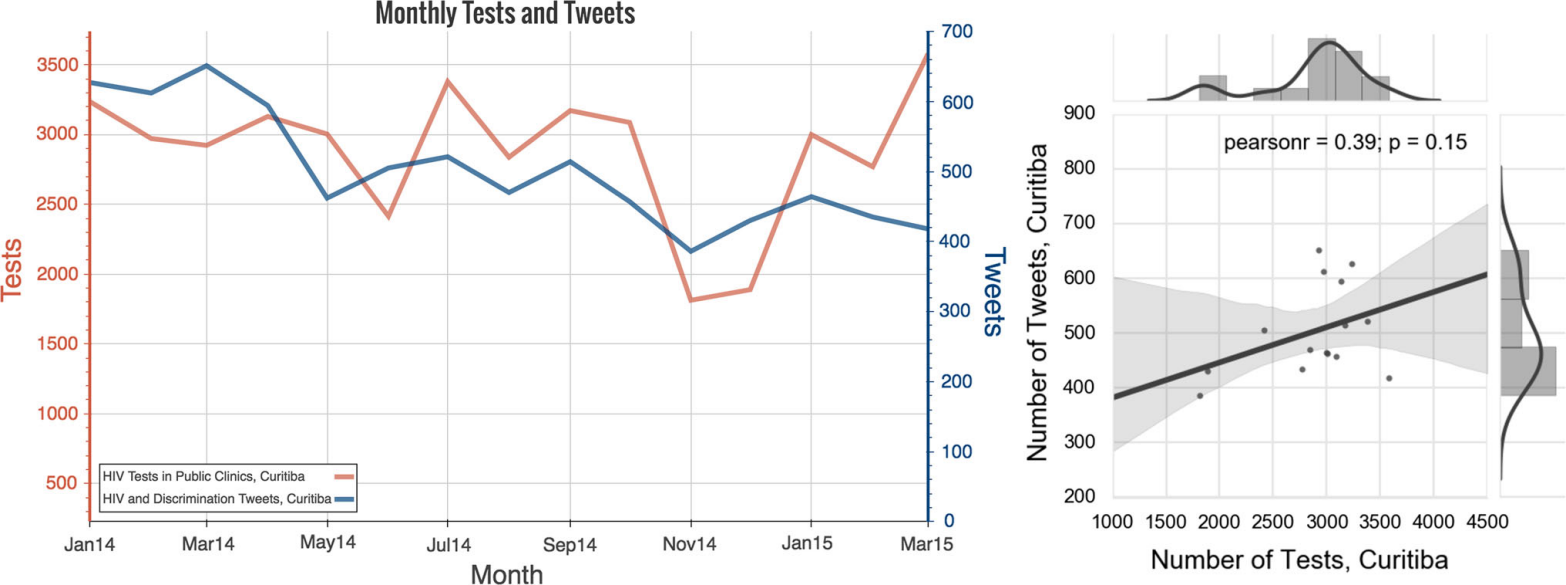
*Left* Time series of monthly number of HIV tests conducted in public clinics in Curitiba during the period studied, January 2014–March 2015, and a number of tweets written about HIV and AIDS and discrimination during the same time period. *Right* Number of tweets graphed as a function of number of HIV tests

Monthly HIV test data from the *Secretaria Municipal da Saúde de Curitiba* (Curitiba’s Health Department) was collected. Tweets geo-located to Curitiba were grouped by month to secure comparability, resulting in two 15-point time-series that were used for a simple correlation study. While the data points used for this study and the results are not relevant enough to extract any definitive conclusions, there is a moderate positive correlation of .39 between monthly number of HIV diagnostic tests and monthly number of tweets (see Fig. 4). It is worth to note that Municipal Laboratory moved premises in November 2014 reporting a temporary reduced number of samples collected during November and December.

**Fig. 5:**
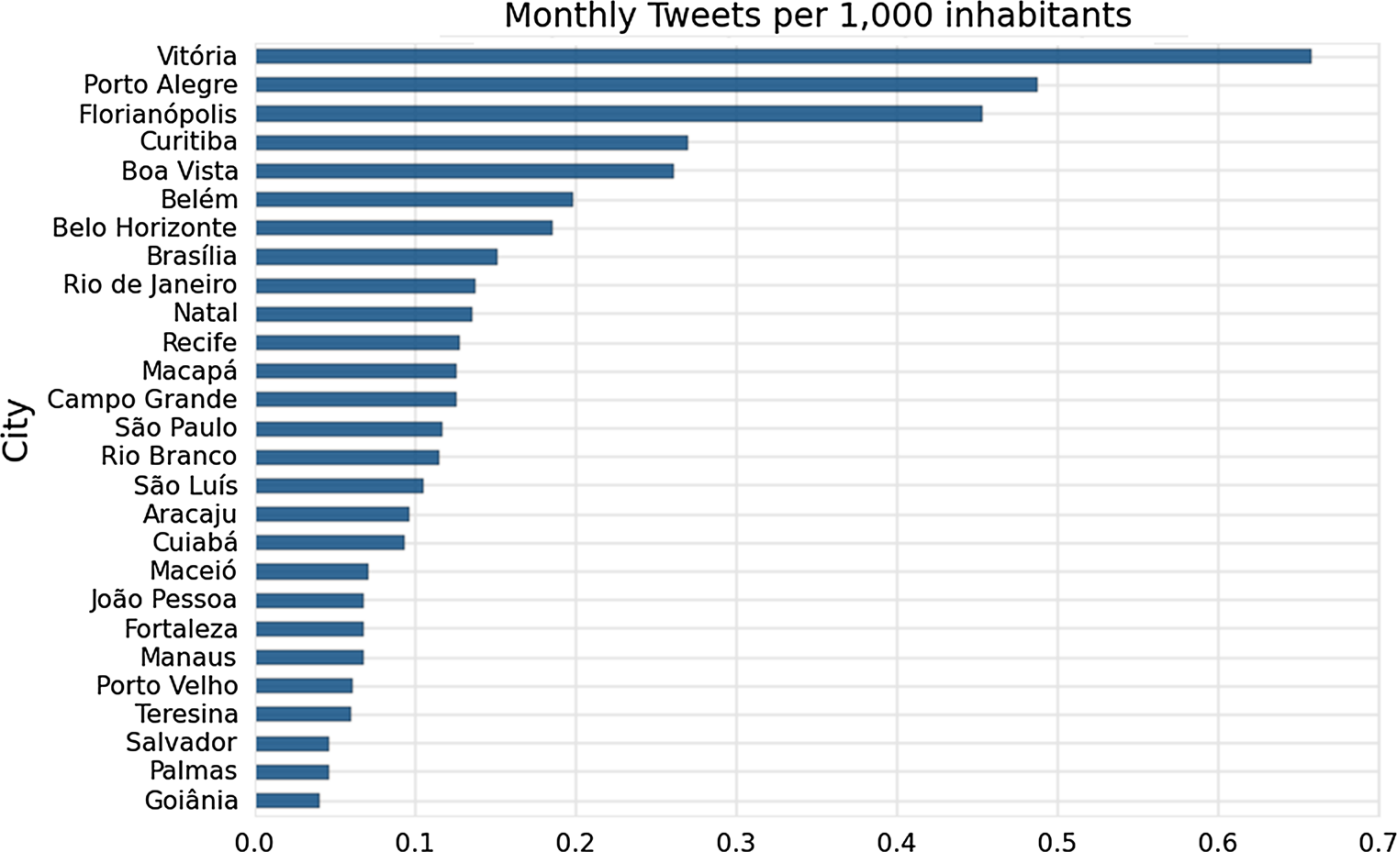
Number of HIV/AIDS and discrimination tweets per month per 1000 inhabitants

In order to provide additional insights and ideas of potential uses of social media data as proxy for HIV monitoring, we proposed an indicatorshowing the number of HIV and AIDS and discrimination tweets per inhabitant (Fig. 5) (population data extracted from [19]). Similar research [15] in US suggests that there is a significant positive relationship between HIV-related tweets and HIV cases. In the absence of HIV testing data, it was not possible to quantify the difference between both rankings. However, the higher number of HIV related tweets are observed predominantly in areas where HIV prevalence is also higher (South and South-East of Brazil), and where urbanization and population density is higher with the exception of Boa Vista and Belém in the northern region of Brazil.

## Discussion

Big data can be used to assess perceptions about public health issues. This study assessed social media data from Twitter to inform communication campaigns to promote HIV testing and reduce discrimination related to HIV and/AIDS or towards key populations to the HIV epidemic, and its potential utility to evaluate such campaigns through HIV testing uptake.

The results of this study suggest that there is a potential to use social networking data for improved messaging in campaigns. However, the validity of social media as a proxy for HIV testing uptake could not be fully assessed.

The main limiting factor to determine whether a clear connection exists between discriminatory or negative messages and uptake of HIV testing was the lack of ground-truth data at same geographical granularity and for the same time periods and frequency. Therefore, further methodological validation would require a large-scale validation process, which is needed to compare social data and clinical data for different geographies and longer term periods, as well as deeper understanding of the bias in social media data [20, 21]. While more traditional surveillance data systems will continue to be crucial in public health, we hypothesize that real-time social media data could serve as a “smoke detector” for negative and discriminatory attitudes and behaviours in the communities and trigger deeper investigations and earlier programmatic response.

Even with the great efforts of developing a detailed taxonomy (1966 keywords and phrases reached after several iterations) for the classification of Tweets, some of them may have been misclassified in the process. The authors noted that in the continuous process of filtering and classifying the tweets, a mechanism needs to be established to review and adjust if any systematic errors in taxonomy are identified—for instance new errors might appear when some of the words included in the taxonomy start to be used by the general population in a different context than ours. Furthermore, the evolution of false positive and false negative rates from iteration to iteration of the taxonomy should be measured. In this study, such errors were not identified nor evaluated.

In using this type of tool for communication and advocacy purposes, practitioners found that the visualization dashboard (potentially public-facing) enables both program managers and civil society to monitor the public reactions towards the campaigns, which facilitates program managers in establishing a more dynamic interaction with the population in promoting health messages and deconstruct misconceptions on HIV related issues. Planned and structured campaigns used in tandem with social media can support other programmatic efforts (HIV prevention and testing) and supportive environment of reduced stigma and discrimination.

Further investments should be made in designing protocols that incorporate new information channels into daily programme operations—e.g., “if the discussion around topic X is emerging, messaging Y should be communicated using Z influencer channels” and establish clear metrics of success based on the transfer function from the digital to the physical world. Such protocols can support positive health seeking behaviour, early diagnosis, and effective treatment outcomes for HIV and other health and social issues. They could be used in early interventions, e.g., such as the use of new media communications around emerging infectious diseases, or in connection with big international or national events that require an effective coordination of health communications and other services. Quick and coordinated communication efforts can influence people’s perceptions and support positive behaviours. Recognizing, valuing and exploiting the potential of new data sources will require to adjust communication strategies and to redesign the way communication teams implement and adapt health campaigns.

## Electronic supplementary material

Below is the link to the electronic supplementary material.

Supplementary material 1 (DOCX 11 kb)
