# Supplementary material for: Social Media Monitoring of Discrimination and HIV Testing in Brazil, 2014–2015
Source: AIDS Behav. 2017 Mar 27;21(Suppl 1):114–20. doi: 10.1007/s10461-017-1753-2 (PMC5515980; doi:10.1007/s10461-017-1753-2)
Supplement: Supplementary file 1 — Supplementary material 1 (DOCX 11 kb) [file 10461_2017_1753_MOESM1_ESM.docx]

# Supplementary Material

## Portuguese Taxonomy

To enable others to continue the work and monitor similar campaigns, we share the Portuguese language taxonomy exactly as used in this project. It is shared as a text file and is written in Datasift’s Curated Stream Definition Language (CSDL).

**Taxonomy**: <http://unglobalpulse.net/discrimination-and-hiv/taxonomy.txt>

**CSDL Documentation** (Nov. 2015): <http://dev.datasift.com/docs/csdl>

# Tweet IDs

To enable reproducibility, we also share the complete set of Tweet IDs used in this study. By using the open Twitter API, it is possible to reproduce the full dataset except any tweets that have since been deleted.

This practice follows Twitter’s Developer Policy, I-6-b (November 2015): "If you provide Content to third parties, including downloadable datasets of Content or an API that returns Content, you will only distribute or allow download of Tweet IDs and/or User IDs."

**Tweet IDs**: <http://unglobalpulse.net/discrimination-and-hiv/tweet-ids.csv>

**Twitter Developer Policy**: <https://dev.twitter.com/overview/terms/agreement-and-policy>

**Examples of Tweet Retrieval Tools**: <https://github.com/digitalmethodsinitiative/dmi-tcat> and <https://github.com/renecnielsen/twitter-diy>

By combining the language-driven topical classification from the taxonomy and the tweets retrievable from the Tweet IDs, it should be possible to reproduce this study completely.
